# Physical parameters of fabrics which contribute in wetness sensation throughout a high-intensity exercise in a warm environment

**DOI:** 10.1186/2046-7648-4-S1-A151

**Published:** 2015-09-14

**Authors:** Florence Agapé

**Affiliations:** 1 Thermal comfort Laboratory, Department of Research and Development, DECATHLON, France

## Introduction

Water transmissibility and diffusion rate of water of a fabric are main parameters that are often used to be the most effective to describe the capacity of a fabric to manage moisture in order to limit wetness sensation. But are they the most relevant parameters to predict wetness sensation throughout the exercise? The purpose of this study was to assess the physical properties of fabrics and physiological responses that predict wetness sensation throughout a high-intensity exercise in a warm environment.

## Methods

Twenty one healthy men performed a 40 minutes run on a treadmill at 12 km.h^-1 ^in a warm environment (25 °C and 50 % rh). Ten short-sleeved t-shirts with different characteristics (thickness, weight, air permeability and moisture management) were tested. Skin temperature was monitored on seven sites of the subjects (chest, lower torso, thigh, calf, upper arm, upper back, lower back) using i-button thermochrons (Dallas Semi-conductor). Whole-body sweat loss and remaining amount of water in the shirts were measured with an electronic scale. Subjects were asked to rate their wetness sensation using a 9 points scale (from 0 "dry" to 8 "extremely wet") and to determine whether these sensations were acceptable or not every five minutes.

## Results

Wetness sensation increased with time (R² = 0.98) and starts to be unacceptable by participant at 15 minutes (96% of acceptability). It's at this time that correlation between wetness sensation and properties of fabric become stronger, particularly, at 20 minutes, wetness sensation had the strongest correlation with properties of fabric. Indeed, wetness sensation was correlated, significantly, with thickness (mm, R² = 0.85), water retention (g.m^-^², R² = 0.70), drying time (min, R² = 0.70) and diffusion rate of water (mm/s, R² = 0.57). At 40 minutes, wetness sensation was still correlated with thickness and drying time.

## Conclusion

Throughout a high-intensity exercise in a warm environment, thickness, retention and drying time are the parameters having the strongest correlation with wetness sensation. They are the only parameters which remain correlated to wetness sensation throughout the activity. These correlations become stronger between 15 and 20 minutes of the exercise, then weaken toward the end. As retention and drying time are strongly correlated to thickness (R ² = 0.90), we can say that the parameter which is mainly correlated to wetness sensation is thickness.

## Discussion

This study shows that the parameters usually described to assess the moisture management capacity of textile and predicting wetness sensation, are not the parameter the most efficient to predict wetness sensation. Indeed, when steady state is reached, water transmissibility can no longer have an impact on water transfer. It's parameters bound to the exchange area of the textile such as the diffusion rate of water then those bound to the retention and the drying performance of the textile.

